# Simplified shielded MEG-MRI multimodal system with scalar-mode optically pumped magnetometers as MEG sensors

**DOI:** 10.1038/s41598-024-77089-z

**Published:** 2024-10-28

**Authors:** Yosuke Ito, Hiroyuki Ueda, Takenori Oida, Takahiro Moriya, Akinori Saito, Motohiro Suyama

**Affiliations:** 1 Department of Electrical Engineering, Graduate School of Engineering, Kyoto University, Kyoto-daigaku Katsura, Nishikyo-ku, Kyoto, 615-8510 Japan; 2 Central Research Laboratory, Hamamatsu Photonics K.K., Hamamatsu, Japan; 3 Global Strategic Challenge Center, Hamamatsu Photonics K.K., Hamamatsu, Japan

**Keywords:** Magnetic resonance imaging, Magnetoencephalography, Biomedical engineering, Imaging and sensing

## Abstract

Magnetoencephalography (MEG) conventionally operates within high-performance magnetic shields due to the extremely weak magnetic field signals from the measured objects and the narrow dynamic range of the magnetic sensors employed for detection. This limitation results in elevated equipment costs and restricted usage. Additionally, the information obtained from MEG is functional images, and to analyze from which part of the brain the signals are coming, it is necessary to capture morphological images separately. When MEG and morphological imaging devices are separate, despite their individual high measurement accuracies, discrepancies in positional information may arise. In response, we have developed a low-field magnetic resonance imaging system that incorporates scalar-mode optically pumped magnetometers with a wide dynamic range and exceptionally high measurement sensitivity as sensors for MEG. Operating at low magnetic fields eliminates the need for superconducting coils in magnetic resonance imaging and the high-performance magnetic shields essential for MEG, promising a substantial cost reduction compared to traditional approaches. We achieved a noise level of about ${16.7}\,\hbox {pT/Hz}^{1/2}$ with a single channel magnetometer, and reached a noise level of ${367}\,\hbox {fT/cm/Hz}^{1/2}$ with a baseline of 1 cm through differential measurements. We employed this system to conduct sequential OPM-based magnetic field measurements and MRI imaging, successfully demonstrating the compatibility of high OPM sensitivity with clear MRI acquisition.

## Introduction

There is a growing need for high-sensitivity magnetic sensors that do not utilize liquid helium, such as high-$T_c$ superconducting quantum interference devices (SQUIDs) and optically pumped magnetometers (OPMs), partly due to the recent global helium shortage. These sensors are particularly being highlighted in the field of biomagnetic measurements, with ongoing advancements in their application to magnetoencephalography (MEG) and magnetocardiography (MCG). On-scalp MEG using these sensors is especially the focus of active research. High-$T_c$ SQUIDs operate with cooling by liquid nitrogen and, while they have lower magnetic field sensitivity compared to low-$T_c$ SQUIDs that use liquid helium, they allow the sensor to be positioned closer to the signal source due to their thinner insulation layers. Additionally, they benefit from the ability to leverage the technology developed for low-$T_c$ SQUIDs. The development of MEG technology incorporating high-$T_c$ SQUIDs is progressing^1^, with commercial applications having commenced.

On the other hand, OPMs can achieve measurement sensitivity comparable to low-$T_c$ SQUIDs without the need for cryogenic substances^2,3^. The use of OPMs in biomagnetic measurements has seen significant advancements in recent years^4–9^. The OPMs’ cryogen-free operation also enables closer proximity to the signal sources, offering the added benefit of acquiring more substantial signals when compared to low-$T_c$ SQUID-based magnetometers. Boto et al. have demonstrated success in acquiring magnetic signals caused by voluntary movements within a magnetic shield using the OPMs along with meticulously calculated compensating coils^5^. Furthermore, Limes and colleagues have successfully measured auditory evoked magnetic fields in a favorable magnetic environment in outdoor suburban settings by differentially measuring the precession frequencies of rubidium spin polarization induced by a powerful pulsed laser^10^. This type of OPMs is known as scalar-mode OPMs, and by employing them, highly sensitive magnetic field measurements can be achieved even in unshielded environments^11,12^. Utilizing the commercial scalar-mode OPMs, Jaufenthaler et al. achieved successful observations of the relaxation of magnetic moments in magnetic nanoparticles, even in an unshielded environments^13^.

MEG and other biomagnetic field measurements provide functional images, and this holds true for the measuremnents with OPMs as well. To perform source localization, it is imperative to co-register it with structural imaging^14,15^. However, imaging modalities like computer tomography (CT) and magnetic resonance imaging (MRI), which yield structural data, cannot be acquired simultaneously with MEG, and in fact, there are few examples of measurements made with the same equipment. CT requires X-ray exposure around the subject, which can introduce radiation risks and limit sensor placement. Furthermore, MRI can disrupt sensor operation due to the presence of strong static magnetic fields, gradient fields, and RF fields. While not yet commercialized, several groups have successfully developed MEG-MRI systems by integrating ultra-low field MRI with MEG based on SQUID technology^16,17^. Developing a multimodal system that combines MEG with structural imaging devices remains a highly complex endeavor.

In recent years, there have been proposals for MRI systems utilizing OPM technology^18–21^, but these have not been explored as multimodal systems alongside MEG. An advantage of OPM-MEG is the ability to customize a flexible sensor array to match the size and shape of a subject’s head^6^. However, this advantage makes the process of overlaying functional and anatomical images, typically done more easily in conventional SQUID-MEG, somewhat challenging. Thus, there is a growing interest in achieving a multimodal system for OPM-MEG that can also capture anatomical images.

In addressing this issue, we propose a multimodal system for MEG by differential measurements with scalar-mode OPMs and low-field (7-mT) MRI with a non-cryogenic pickup coil and normal conducting coil sets in a simplified magnetic shield. In comparison to using OPM as an MRI detector, pickup coils exhibit superior characteristics in terms of receiving bandwidth and stability. Therefore, in this study, OPM was employed for MEG detection, while the pickup coil was utilized for MRI detection. Frequency range of scalar-mode OPMs is determined by the pump-probe cycle, making it capable of measuring signals up to kHz^22^. Moreover, the scalar-mode OPMs, characterized by their wide dynamic range, facilitate high-sensitivity measurements even within a simplified magnetic shielding environment. On the other hand, in the case of MRI, the economical normal conduction coil sets allow for easy switching of the static magnetic fields, ensuring that it does not hinder the operation of the scalar-mode OPMs during MEG measurements. In our developed 7-mT MRI system, Larmor frequency is around 300 kHz, providing sufficient detection sensitivity even with the non-cryogenic pickup coil^23^.

This study describes the development of a scalar-mode OPM module enabling differential measurements and its integration into a simplified magnetic shielded low-field MRI system, including experimental assessments of its performance.

## Methods

### Scalar-mode optically pumped magnetometer module

**Fig. 1:**
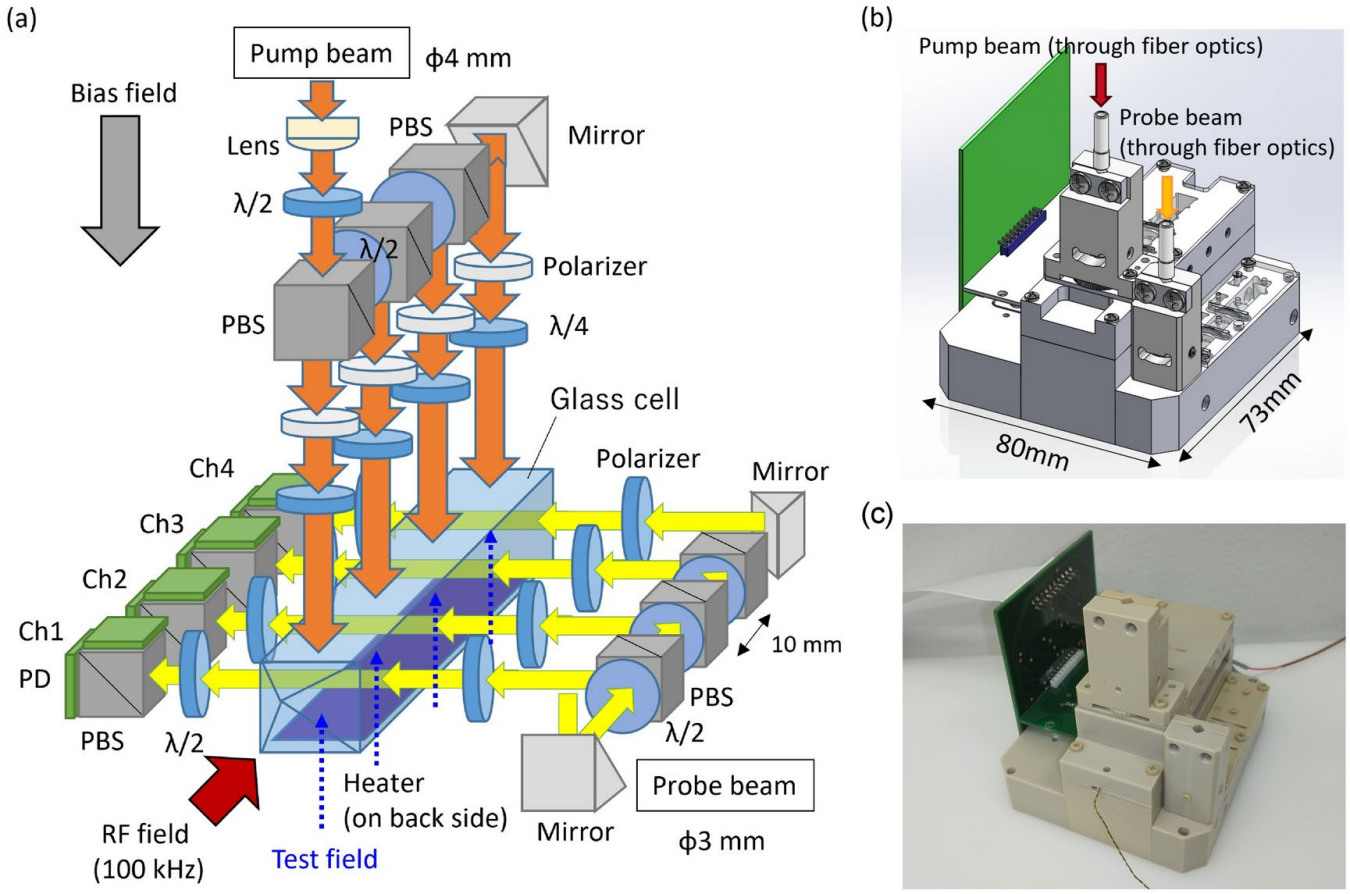
4-ch scalar-mode OPM module. (**a**) Schematic drawing, (**b**) overview, and (**c**) photograph of 4-ch scalar-mode OPM module. The pump and probe beams were introduced to the module via fiber optics, undergo polarization adjustment by waveplates, and then were distributed to four points within a glass cell by polarizing beam splitters (PBSs). The beams passing through the glass cell were detected by polarimeters consisting of a PBS and two photodiodes (PDs). The glass cell, which contains potassium, helium, and nitrogen, was heated by an electric heater installed on one side of the glass cell.

Figure 1a illustrates a schematic diagram of the 4-ch scalar-mode OPM module that we have developed. The pump and probe beams were introduced to the module via fiber optics, undergo polarization adjustment by waveplates, and then were distributed to four points within a glass cell by polarizing beam splitters (PBSs). The diameter of the pump beam was 4 mm, and that of the probe beam was 3 mm. The beams passing through the glass cell were detected by polarimeters consisting of a PBS and two photodiodes (PDs). The glass cell, which contains potassium, helium, and nitrogen, was heated up to 120 °C by an electric heater installed on one side of the glass cell. The overview of the module is shown in Fig. 1b. The footprint of the module was 80 mm $\times 73$ mm, within which four channels were arranged linearly. Each channel was positioned at 10 mm apart, allowing for the calculation of tangential differential outputs by subtracting their respective signals. Figure 1c displays a photograph of the module. The module is fabricated from polyetheretherketone (PEEK). The wire for the heater was introduced from the opposite side of the fiber optics ports. In this setup, the OPM module is placed between biplanar bias and RF magnetic field coils, both integrated into a PCB that is not depicted in the figure.

Our scalar-mode OPM scheme is based on the methodology presented in Ref.^24^. In brief, circularly polarized pump beam is pulsed into the sensor cell, inducing spin polarization. This spin polarization is then tipped into a plane orthogonal to the bias magnetic field by an RF pulse. Once the RF pulse is turned off, the polarized spins precess and gradually relax back to alignment with the bias field. The precession frequency depends on the strength of the bias field, and in this study, we set the bias field $\left| \varvec{B}_\text {bias}\right|$ such that the precession frequency is 100 kHz, as determined by following equation:

1 $$\begin{aligned} {f = \frac{\gamma _e \left| \varvec{B}_\text {bias} + \varvec{B}_\text {e}\right| }{2 \pi q},} \end{aligned}$$

where *f* is the presession frequency, $\varvec{B}_\text {e}$ is the external magnetic field, $\gamma _e$ is the gyromagnetic ratio of the isolated electron, and *q* is the slow-down factor.

This behavior can be detected by passing linearly polarized probe beam through the sensor cell. If an external magnetic field is applied, the precession frequency shifts accordingly, allowing the magnitude of the external magnetic field to be calculated based on the deviation from the reference frequency. For this OPM, the bandwidth when detecting magnetic fields significantly weaker than the bias field is determined by half of the sampling frequency, specifically half the frequency at which the pump beam, RF pulses, and data acquisition cycles are repeated.

**Fig. 2:**
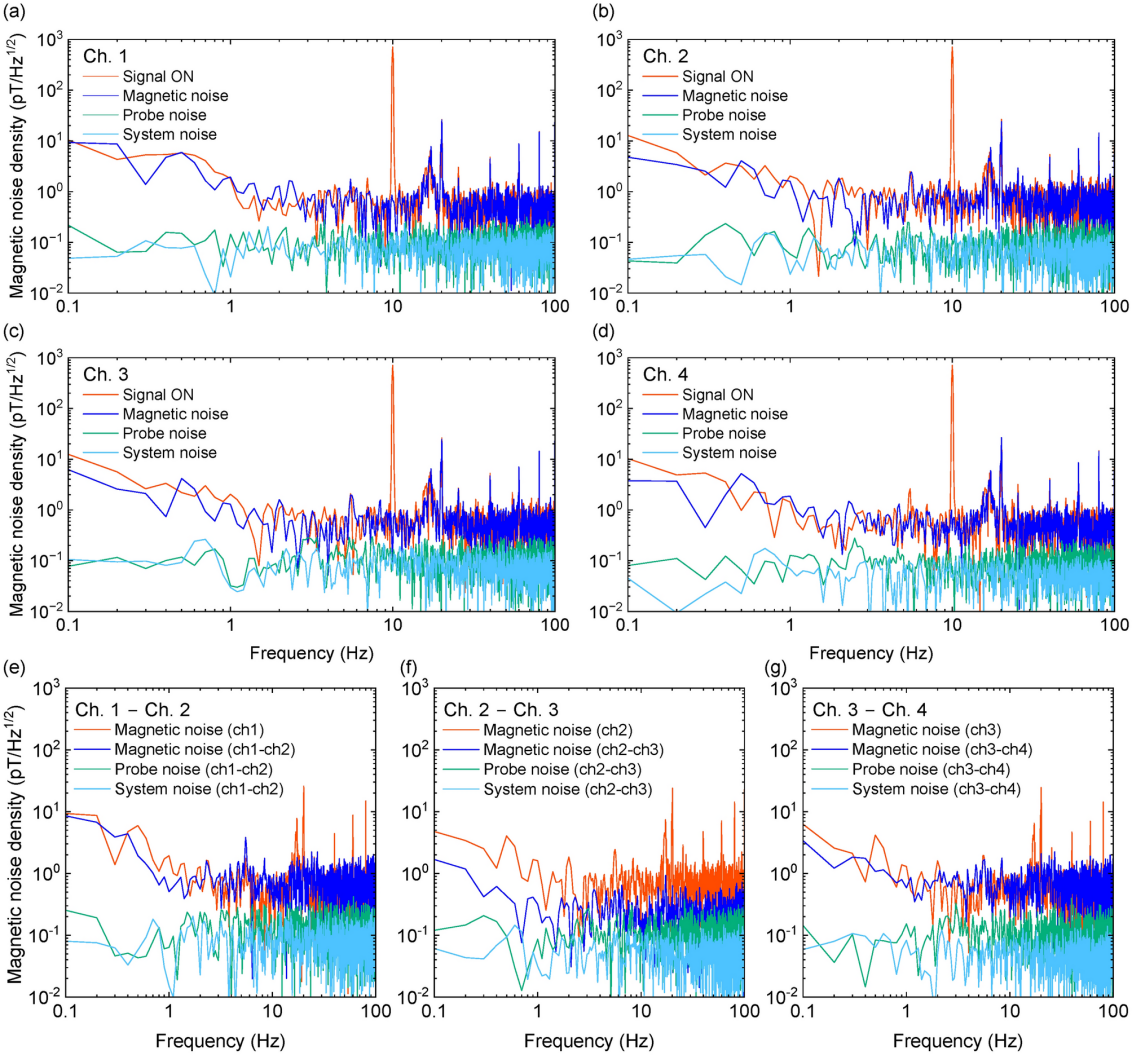
Potential of the 4-ch scalar-mode OPM module. Magnetic noise denisty at (**a**) Ch. 1, (**b**) Ch. 2, (**c**) Ch. 3 and (**d**) Ch. 4 of 4-ch scalar-mode OPM module in magnetic shield. The reference field was 10-Hz sinusoidal wave with an amplitude of 318 pT. Probe noise and system noise were estimated from noise level of probe beam noise and amplifier noise. Magnetic noise denisty based on differential signals of each pair of two channels (**e**) Ch. 1 − Ch. 2, (**f**) Ch. 2 − Ch. 3 and (**g**) Ch. 3 − Ch. 4 of 4-ch scalar-mode OPM module in magnetic shield.

The magnetic noise density at each channel in a magnetic shield is shown in Fig. 2a–d. The shielding factor of the magnetic shield was > 10^4^ at 10-Hz. We applied spatially uniform 10-Hz sinusoidal wave as a reference field. The measurement time was 10 s and the sampling rate was 200 Hz. The magnetic noise density was 560, 669, 580, and 562 fT/Hz^1/2^ at Ch. 1, 2, 3, and 4, respectively. Several peaks observed above 20 Hz in Fig. 2a–d are attributed to harmonics of the 60 Hz commercial power supply noise, which have been recorded due to aliasing. Additionally, the gradual peak between 10 and 20 Hz is likely caused by environmental factors such as the operation of the air conditioning system and the resulting vibrations in the fiber optics. While the aliasing effect is almost entirely mitigated by the use of differential measurements, the peaks in the 10–20 Hz range remain partially present even with the differential measurements. This indicates that further efforts to enhance system stability will be necessary in the future.

In order to distinguish between the impact of magnetic field noise and system-inherent noise, we evaluated the influence of both probe noise and system noise (electrical noise from the detector circuit). The noise measured when the pump beam was turned off was superimposed on a sinusoidal signal, and the frequency estimation error derived from Eq. (1) was regarded as the probe noise. In the same way, the noise observed with the probe beam off was used to estimate the system noise. The estimated probe beam noise was 69, 55, 143, and ${102}\,\hbox {fT/Hz}^{1/2}$ at Ch. 1, 2, 3, and 4, respectively. The probe beam noise was less than the magnetic field noise, therefore the magnetic noise still remained. Figure 2e–g shows magnetic noise density based on differential signals of each pair of two channels. The magnetic noise density was ${560}\,\hbox {fT/cm/Hz}^{1/2}$ at Ch.1 − Ch.2, ${154}\,\hbox {fT/cm/Hz}^{1/2}$ at Ch.2–Ch.3, and ${564}\,\hbox {fT/cm/Hz}^{1/2}$ at Ch.3–Ch.4. For Ch. 2–Ch. 3, there was a substantial enhancement in measurement sensitivity, whereas there was less apparent improvement in other cases. This could be attributed to the suppression of the impact of non-uniform magnetic fields in the central region of the OPM module. Conversely, as one deviated from the central region, the impact of non-uniform environmental magnetic fields became more prominent.

### MRI system

**Fig. 3:**
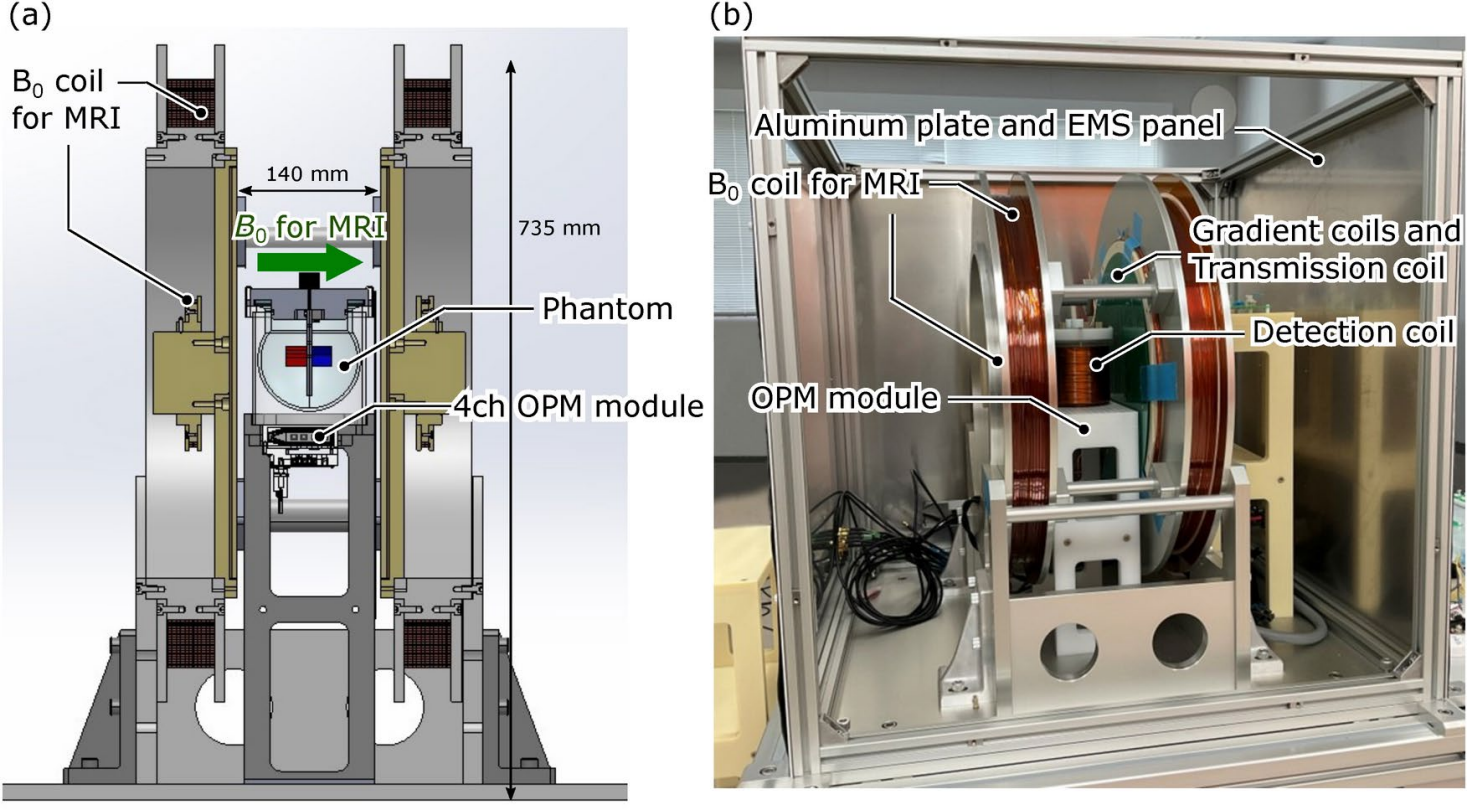
Developed 7-mT MRI system with the 4-ch scalar-mode OPM module. (**a**) Schematic drawing and (**b**) photograph of MRI system. As illustrated in (**b**), The $B_0$ coil for MRI consists of two pairs of coils, ensuring magnetic field homogeneity within the measurement area. The 4-ch scalar-mode OPM module was placed under the phantom, which was surrounded by a detection coil, in the coil set consisting of a transmission coil and gradient coils. The coil set was also present on the opposing surfaces, with the phantom sandwiched in between. The MRI system was surrounded by aluminum plates and EMS panels.

Figure 3a depicts a schematic diagram of an MRI apparatus with the 4-ch scalar-mode OPM module developed by our group. The OPM module was positioned beneath the phantom, which was surrounded by a detection coil, enclosed between the coils for the static magnetic field $B_0$ and the coil set for the transmission and gradient magnetic fields. The strength of the static field for MRI was 7 mT, and the resonance frequency determined by this field was approximately 300 kHz. An important point to note here is that the gyromagnetic ratio of protons differs significantly from that of the OPM. As a result, the value of $\left| \varvec{B}_\text {bias}\right|$ required for OPM operation is markedly different from the value of $B_0$ required for MRI. Therefore, during OPM operation, no current was applied to the MRI coil set, and $\varvec{B}_\text {bias}$ is applied instead. The photograph of this system is presented in Fig. 3b. The entire system was enclosed by aluminum plates and 5-layer EMS panels (EMSPLM05, Medical-aid Co., Ltd.), and the effect of the EMS panels reduces magnetic noise in the frequency range below 100 Hz to approximately 1/5. While the shielding performance is about 1/2000 compared to shields commonly used for MEG, it allows for cost-effective production.

For MRI imaging, a spin-echo sequence was employed^21^, and the imaging parameters are detailed in Table 1. The field of view (FoV) represents the size of the MRI image. In this case, we targeted a region of $96\times 96\times 96 \,\hbox {mm}^3$ with a voxel size of $3\times 3\times 3\, \hbox {mm}^3$. The repetition time (TR) is the time between the excitation pulse and the next excitation pulse, which was set to 300 ms in this study. The echo time (TE) is the time from the excitation pulse to the acquisition of the signal, and in this case, it was set to 25 ms. The flip angle (FA) refers to the angle at which the proton magnetization is tipped from the direction of the static magnetic field by the excitation pulse, and here it was set to ${90}^{\circ }$. The bandwidth (BW) represents the frequency range allocated per pixel, which in this case was 100 Hz per pixel. The number of excitations (NEX) corresponds to the number of signal averages, and in the case of NEX = 8, the measurement signal is averaged over 8 acquisitions. The scan time at NEX = 1 was approximately 5 minutes, while at NEX = 8, it was about 41 minutes, and at NEX = 16, it was approximately 82 minutes.

**Table 1:** MR imaging parameters employed in experiments.

Prameter	Value
FoV	$$96\times 96\times 96$$$$\hbox {mm}^3$$
Matrix	$32\times 32\times 32$
TR	300 ms
TE	25 ms
FA	${90}^{\circ }$
BW	100 Hz/pixel
NEX	1, 8, 16

### Phantom

**Fig. 4:**
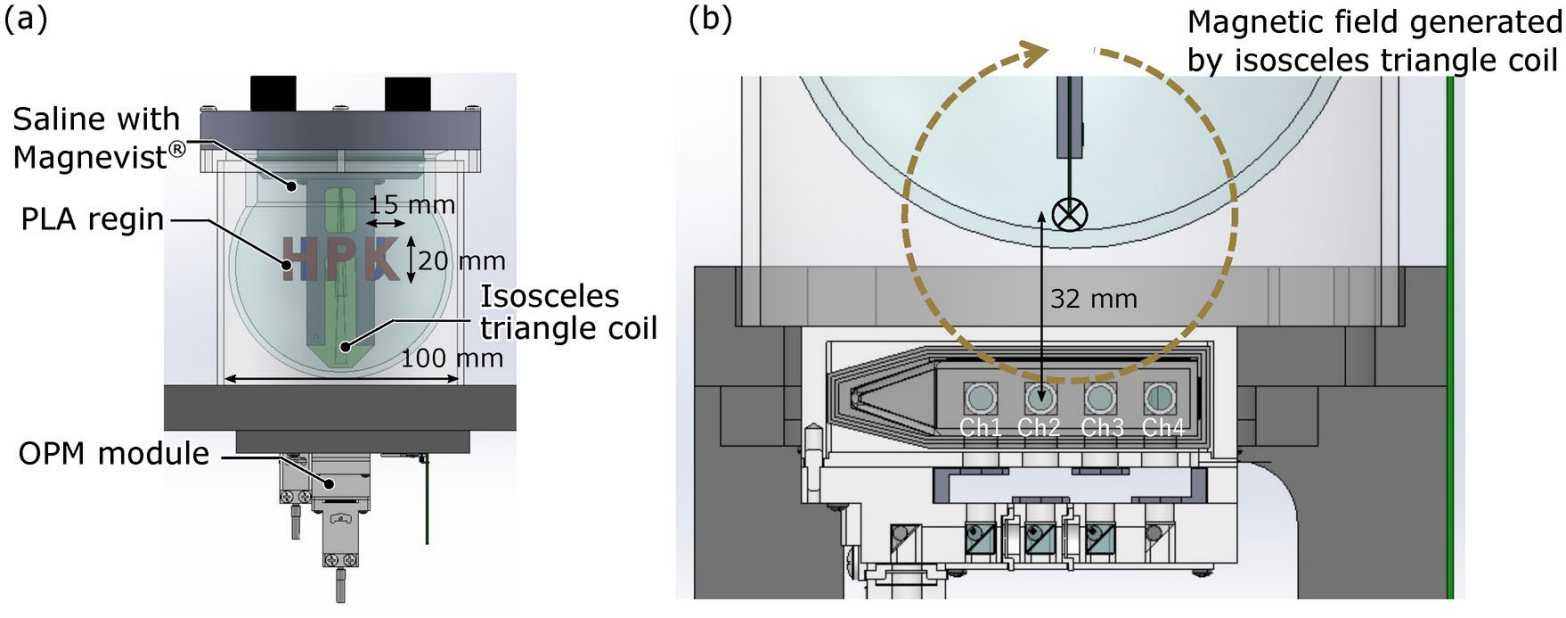
Phantom with objects of ‘HPK’ and ‘KU’, and an isosceles triangle coil. (**a**) Schematic drawing of phantom utilized in the measurements and (**b**) relationship in position between magnetic field generated by the isosceles triangle coil and the OPM module.

Figure 4a illustrates an illustration of the phantom used in the measurements for the OPM performance. The phantom was filled with saline solution supplemented with 1 mM of Magnevist^®^. Magnevist^®^ is an MRI contrast agent, and here, it was added to adjust the T1 and T2 relaxation times of the saline solution to values closer to those of biological tissues. The T1 and T2 relaxation times of the solution were 94 ms and 191 ms, respectively. The phantom contained objects ‘HPK’ and ‘KU’ made of PLA resin, positioned on either side of an ‘H’-shaped frame. The size of each character was 20 mm in height, 15 mm in width, 20 mm in thickness. Additionally, an isosceles triangle coil was positioned beneath the phantom to align with its base^25^. As depicted in Fig. 4b, the distance between the base of the coil and the measurement region of the OPM module was 32 mm, ensuring that the coil was placed directly above channels 2 and 3 of the OPM module. The isosceles triangular coil has the base of 5 mm, and the other two sides are in close proximity. Consequently, the magnetic fields created by the currents along these two sides are nearly canceled out, leaving the base to function as a current dipole. This current dipole is designed to simulate the neural currents within the cerebral cortex, and it was constructed based on the distance from the scalp to the cortex.

## Results

### Sensitivity of 4-ch scalar-mode OPM module

**Fig. 5:**
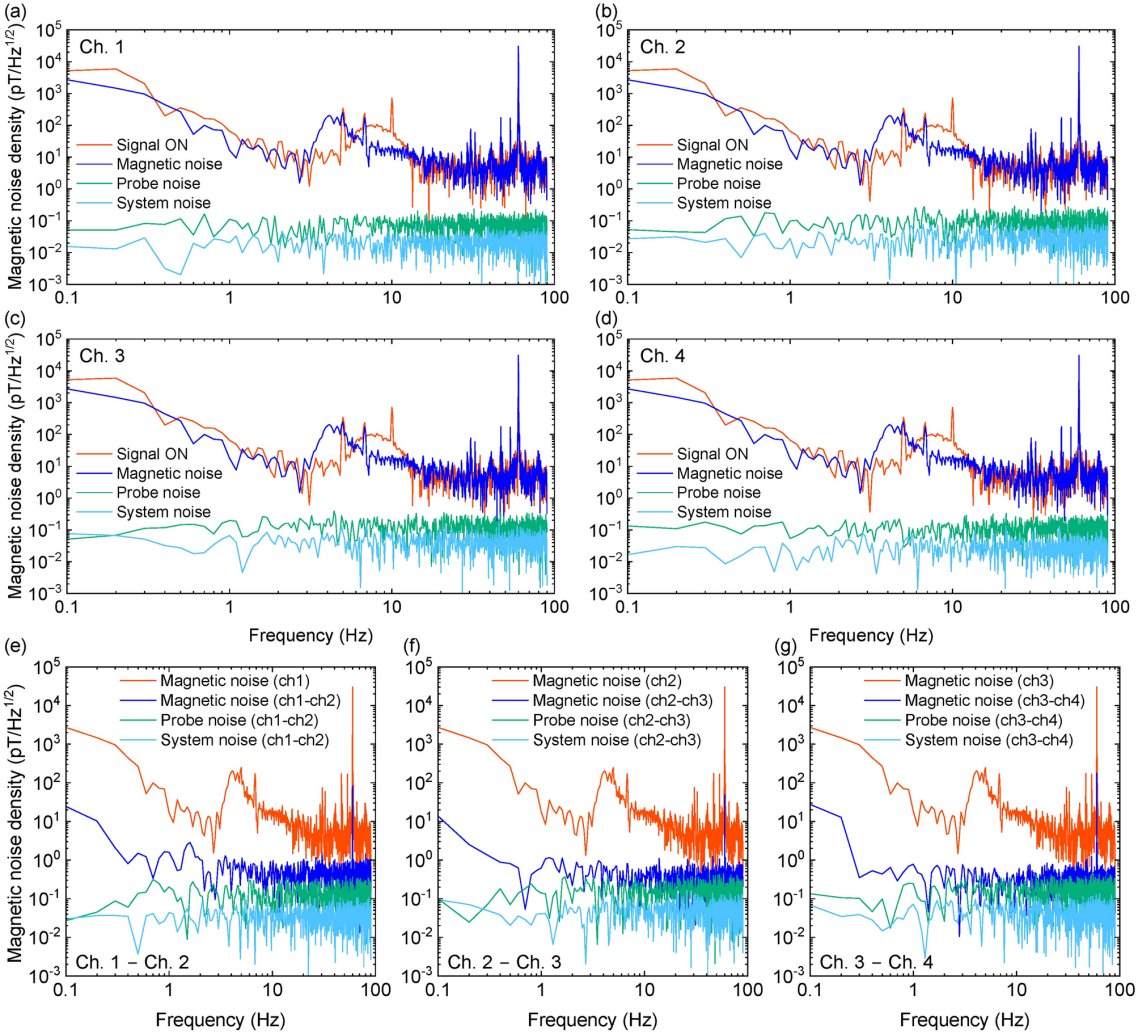
Magnetic noise measurements with the scalar-mode OPM installed in the MRI system. Magneitc noise density at (**a**) Ch. 1, (**b**) Ch. 2, (**c**) Ch. 3 and (**d**) Ch. 4 of 4-ch scalar-mode OPM module. The reference field was 10-Hz sinusoidal wave with an amplitude of 318 pT. Probe noise and system noise were estimated from noise level of probe beam noise and amplifier noise. Magneitc noise density based on the differential signals of each pair of two channels (**e**) Ch. 1–Ch. 2, (**f**) Ch. 2–Ch. 3 and (**g**) Ch. 3–Ch. 4 of 4-ch scalar-mode OPM module.

Figure 5a–d shows magnetic noise density of each channel of the 4-ch scalar-mode OPM module. We applied spatially uniform 10-Hz sinusoidal wave with an amplitude of 318 pT as a reference signal. The measurement time was 10 s and the sampling rate was 180 Hz. The magnetic noise levels at 10 Hz were consistently about 16.7 $\hbox {pT/Hz}^{\mathrm {1/2}}$ across all channels. In all channels, a gradual peak was detected at around 5–15 Hz when the reference signal was on and at around 3–15 Hz when the reference signal was off. These peaks were absent in the magnetic shield measurements shown in Fig. 2, and the change in frequency band and shape when toggling the reference signal, along with their elimination during differential measurements, strongly suggest that the peaks are likely due to magnetic noise from the external environment. However, the exact source of the noise could not be identified. While further detailed investigation is necessary, it is believed not to pose a significant issue since it vanishes with differential measurement.

In terms of probe noise derived from probe beam, it varied significantly across channels, measuring 69, 55, 143, and 102 $\hbox {fT/Hz}^{\mathrm {1/2}}$ for channels 1, 2, 3, and 4, respectively. This variation was attributed to the uneven intensity of probe beam in each channel and the distinct paths taken by each probe beam. Furthermore, the system noise, calculated from amplifier noise, measured 24, 22, 54, and 34 $\hbox {fT/Hz}^{\mathrm {1/2}}$ for channels 1, 2, 3, and 4, respectively, with channel 3 displaying slightly elevated values, but overall comparable magnitudes.

Figure 5e–g presents magnetic noise density of the outputs obtained by differencing each pair of two channels. Since the sensitivity direction of the OPM module aligns with the vertical direction of the channel arrangement, taking the difference of each channel results in measuring the tangential difference as the vertical component along the channel array. Magnetic noise was effectively subtracted by the differential measurements. The magnetic noise was ${538}\,\hbox {fT/cm/Hz}^{1/2}$ at Ch.1–Ch.2, ${367}\,\hbox {fT/cm/Hz}^{1/2}$ at Ch.2—Ch.3, and ${261}\,\hbox {fT/cm/Hz}^{1/2}$ at Ch.3–Ch.4. The noise level at Ch.1–Ch.2 closely resembled the values measured within the magnetic shield. This suggests that even with a simple shield, the impact of spatially uniform environmental magnetic fields can be adequately mitigated through differential measurements.

**Fig. 6:**
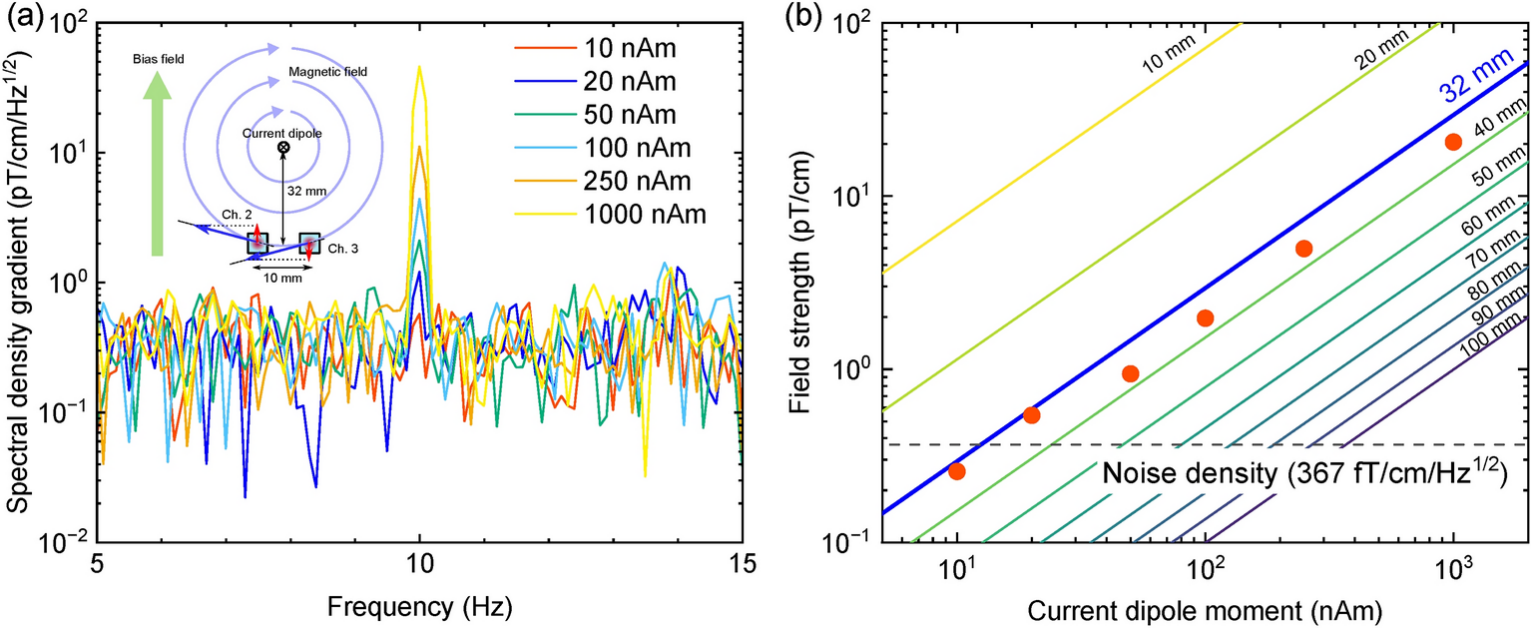
Current dipole moment measurements with the scalar-mode OPM. (**a**) FFT spectra of differential measurements at Ch.2–Ch.3 for the magnetic field generated by the isosceles triangle coil. A sinusoidal current of 10 Hz was applied to the isosceles triangle coil, demonstrating measurements for current dipole magnitudes of 1000 nAm, 250 nAm, 100 nAm, 50 nAm, 20 nAm, and 10 nAm formed by its base. (**b**) Field strength as a function of current dipole moment. The background solid lines incdicate field strength depending on distance between the current dipole and sensing region calculated by Biot-Savart law. Since the tangential component of the magnetic field produced by the current dipole is nearly identical in two channels and cancels out, the magnetic field strength measured in this differential setup reflects the difference in the radial component.

Figure 6a shows FFT spectra of differential measurements at Ch.2–Ch.3 for the magnetic field generated by the isosceles triangle coil. A sinusoidal current of 10 Hz was applied to the isosceles triangle coil. Regarding its base as the current dipole, the magnitude was varied from 1000 to 10 nAm. The peak at 10 Hz can be observed from 1000 to 20 nAm, but not observed with 10 nAm. In this region, the tangential component of the magnetic field generated by the current dipole is nearly equal in two channels and is thus canceled out. As a result, the magnetic field strength obtained from this differential measurement corresponds to the difference in the radial component.

Figure 6b shows field strength as a function of a current dipole moment. In our system, noise density was estimated as ${367}\,\hbox {fT/cm/Hz}^{1/2}$, therefore the detectable current dipole moment was about 15 nAm. The backgound solid lines indicated field strength of the verical magnetic fields at the OPM module depending on distance between the current dipole and sensing region calculated by Biot-Savart law. The experimental data closely matched the calculated results for a scenario where the current dipole and sensing area were separated by 32 mm; however, the magnetic field intensity was consistently lower than expected along the 32-mm line. This discrepancy was likely due to the actual distance between the current dipole and the sensing area being a few millimeters greater than 32 mm. Additionally, when measuring current dipole moment of 100 nAm with this OPM, it is anticipated that measurements can be taken up to approximately 60 mm away from the current dipole. Given that the distance from the sensing area to the sensor module surface is about 12 mm, signal acquisition is estimated to be feasible up to approximately 48 millimeters from the scalp. This result indicated that this OPM module possessed performance capabilities withstanding magnetoencephalography, because the strength of equivalent current dipoles caused by human brain activities is estimated to be 10–100 nAm^26^.

### MR imaging

**Fig. 7:**
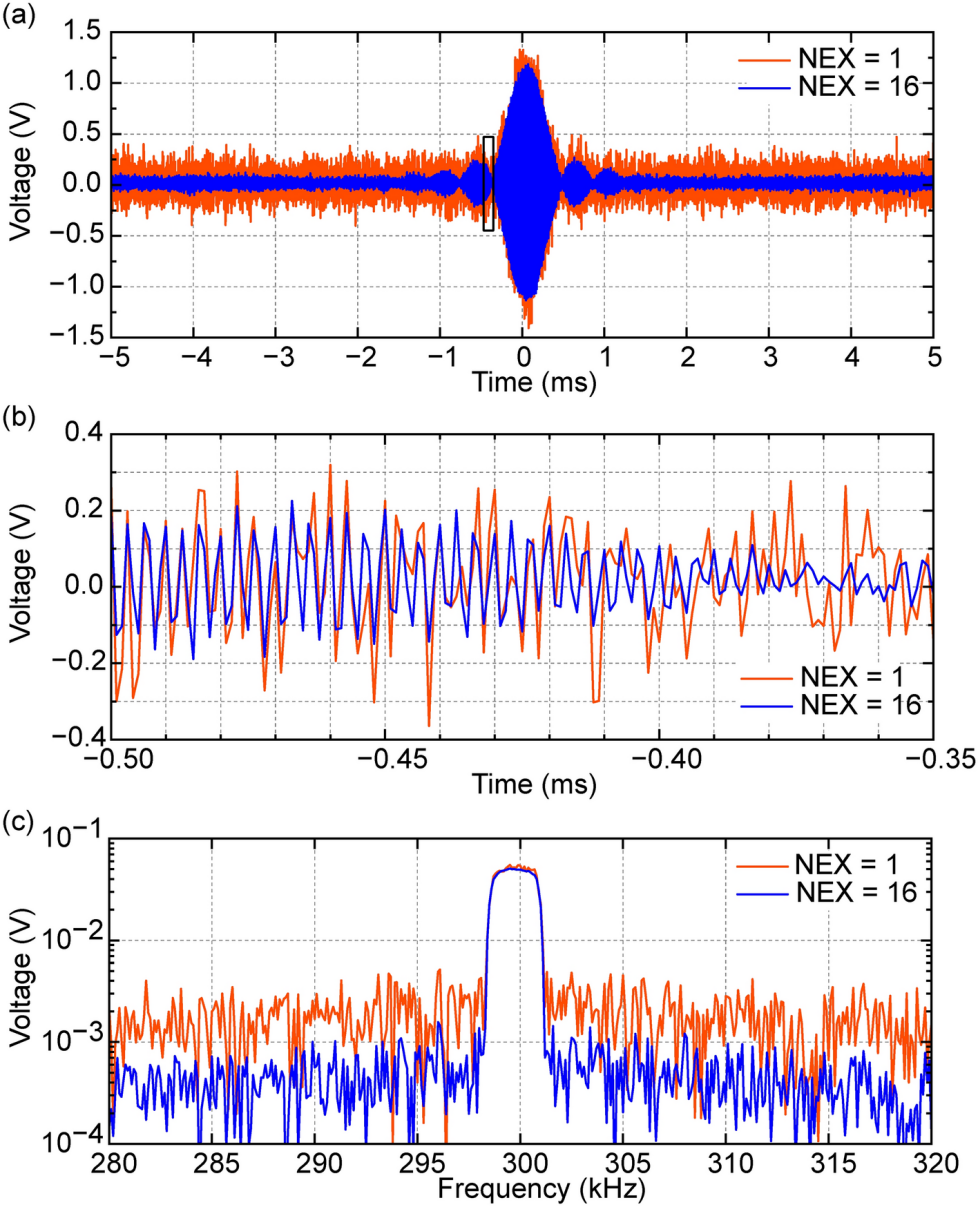
Spin echo signals of the MRI system. (**a**) Time evolution of spin echo signals from a phantom with an absence of internal objects in the absence of phase encoding and slice encoding with NEX = 1 and NEX = 16. (**b**) An enlarged view of the black frame in (**a**), where amplitude-modulated signals at approximately 300 kHz were observed. (**c**) FFT spectra of the spin echo signals.

Figure 7a shows spin echo signals from a phantom with an absence of internal objects and in the absence of phase and slice encoding with NEX = 1 and NEX = 16. Figure 7b is an enlarged view of Fig. 7a and FFT spectra of spin echo signals are shown in Fig. 7c. Amplitude-modulated signals at approximately 300 kHz, which is Larmor frequency of the static field of 7 mT, was observed in Fig. 7b,c. The noise level was roughly estimated to be about one-fourth lower in NEX = 16 compared to NEX = 1, indicating the presence of additive effects.

**Fig. 8:**
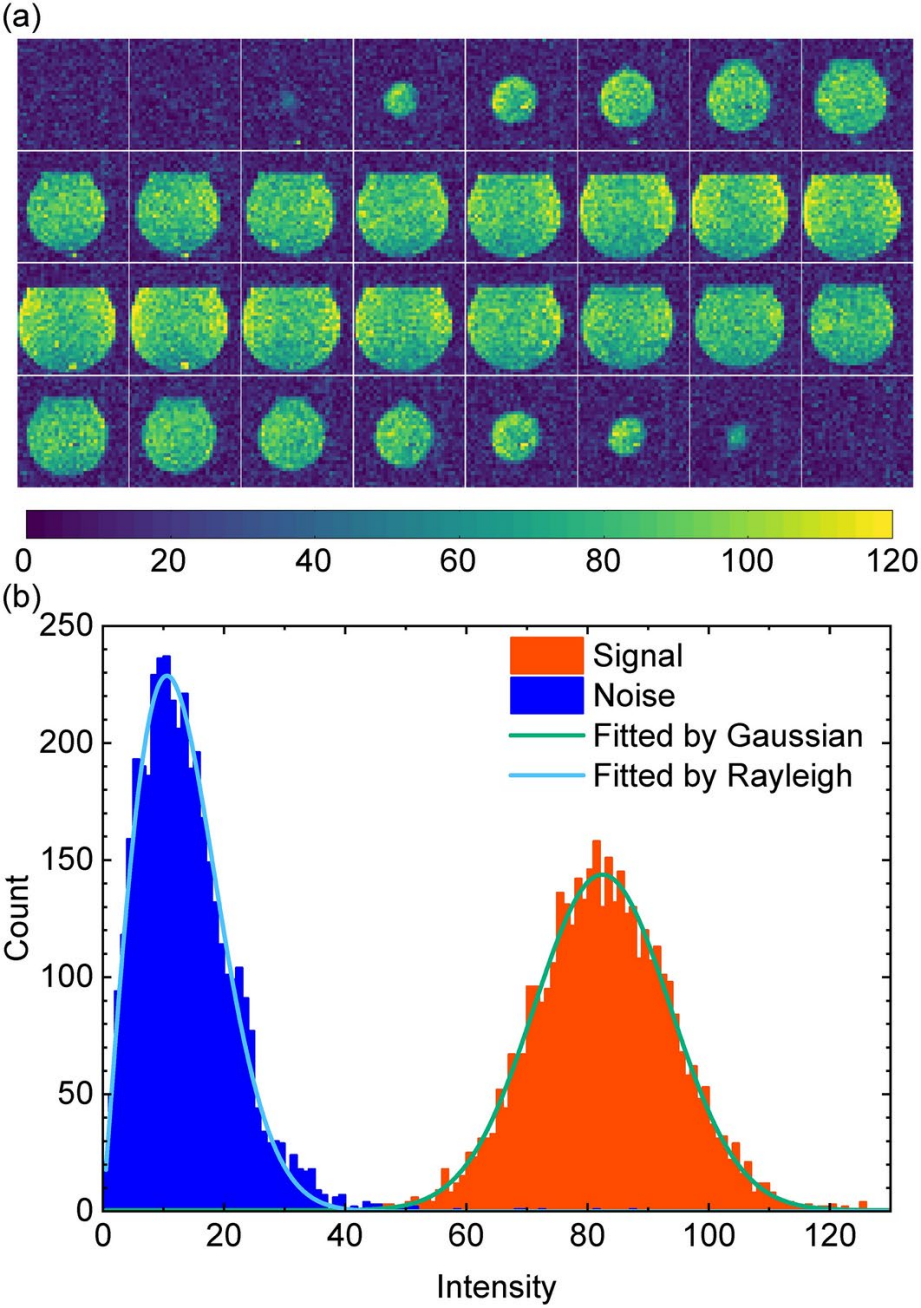
Evaluation of images with the MRI system. (**a**) Reconstruction images of a phantom with an absence of internal objects. (**b**) Histograms of signal and noise domains. Signal and noise domains were defined as the central region of the phantom ($16\times 16\times 16$ voxels) and $8\times 8\times 8$ voxels at each of the 8 corners in the imaging region. The solid lines represent distribution curves of histrograms of signal and noise domains fitted by Gaussian and Rayleigh distribution functions, respectively.

Figure 8a shows reconstruction images of the phantom with an absence of internal objects acquired by applying phase encoding and slice encoding and imaging parameters in Table 1. In this case, we set the NEX to 8. Since the voxel size is $3\times 3\times 3\, \hbox {mm}^3$, the thickness of each image is 3 mm, and the size of one pixel is $3\times 3\, \hbox {mm}^3$. Figure 8b shows histograms of signal and noise domains of the reconstructed images at NEX = 8. The signal and noise domains were defined as the central region of the phantom ($16\times 16\times 16$ voxels) and $8\times 8\times 8$ voxels at each of the 8 corners in the imaging region. The histrograms of signal and noise domains were fitted by Gaussian and Rayleigh distribution because it is well known that the image intensity of MR images follows Rician distribution^27^: it can be approximated by Rayleigh distribution in low intensity regions and by Gaussian distribution in high intensity regions. Therefore, the histograms of signal and noise domains were fitted by Gaussian and Rayleigh distribution.

The mode and the standard deviation (SD) of the intensity at the signal domain were $82.44 \pm 0.12$ and $11.23 \pm 0.14$, and those of the noise domain were $13.23 \pm 0.08$ and $4.53 \pm 0.03$, respectively. The values following the ± symbol represent the standard errors of the fitting. The signal-to-noise (SNR) calculated as the ratio of the mode of the intensity at the signal domain and the noise domain was $6.23 \pm 0.05$, and that calculated as the average intensity divided by the SD of the signal domain was $7.34 \pm 0.10$. According to Rose model, SNR per pixel should be larger than 5 to discriminate signal from noise^28^. Considering 99% confidence interval of the signal domain, the SNR per pixel of the signal domain was larger than 7.8. It was found that artifacts and noise at specific frequencies were quite minimal.

**Fig. 9:**
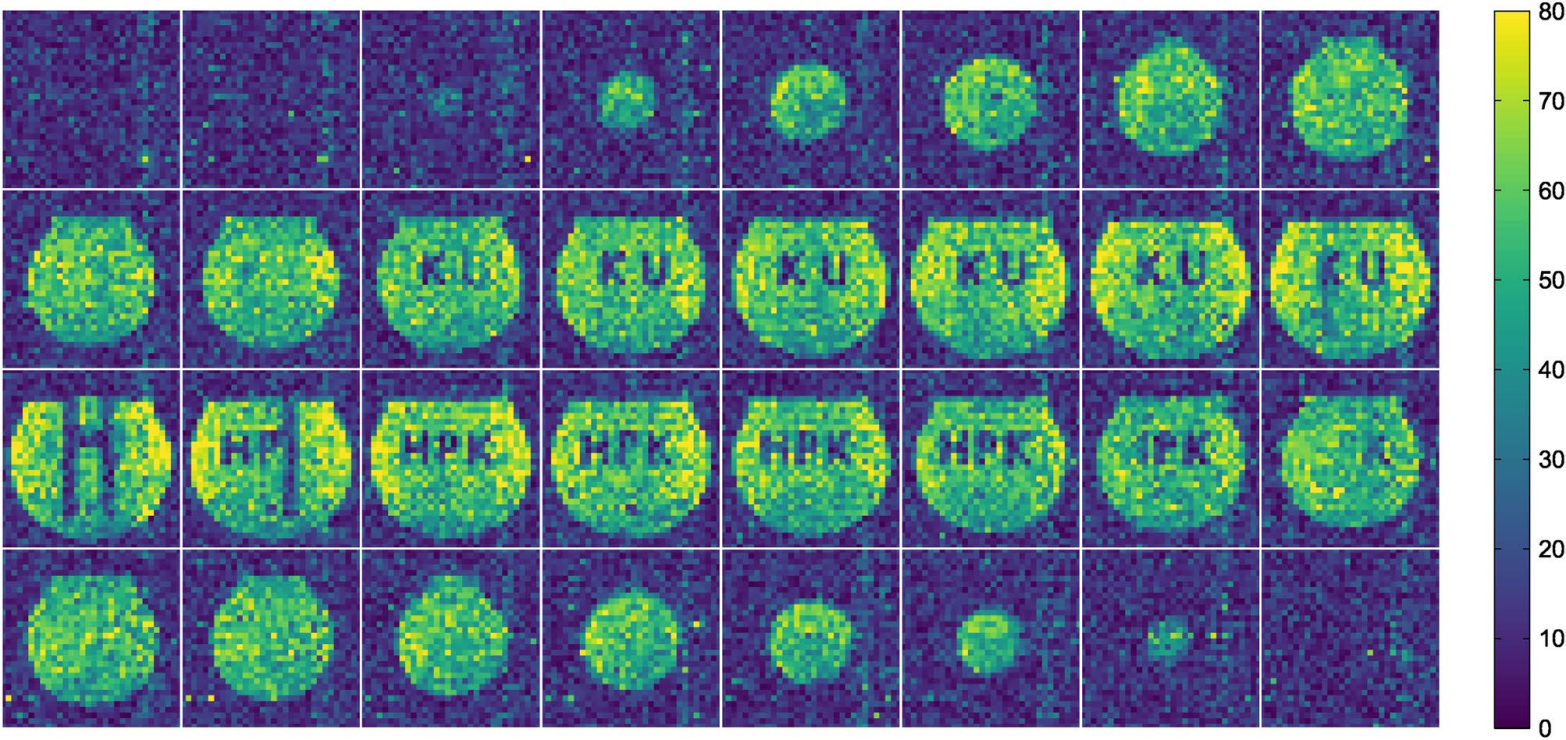
Reconstruction images of 32 different slices. The objects ‘HPK’ and ‘KU’ were observed on different slices.

Using the phantom illustrated in Fig. 4 as the target object, MR signals were acquired by applying phase encoding and slice encoding and imaging parameters in Table 1 with NEX = 8. The reconstructed images are presented in Fig. 9. Since the voxel size is $3\times 3\times 3 \,\hbox {mm}^3$, the thickness of each image is 3 mm, and the size of one pixel is $3\times 3\, \hbox {mm}^3$. The images were obtained in 32 different slices, with the top-left image representing the deepest slice shown in Fig. 4, and moving to the right shows progressively more anterior slices. In Fig. 4, the characters ‘KU’ were located on the reverse side of the characters ‘HPK’, with an ‘H’-shaped frame in between. As the slice transitions, a noticeable interchange of these characters appeared. Starting from the third column from the left in the second row, the ‘KU’ letters can be observed. In the first and second columns of the third row, the ‘H’-shaped frame is visible, and from approximately the second-to-last image in the third row, the ‘HPK’ letters are seen. Considering that each letter is 20 mm thick and the slice thickness is 3 mm, these results are reasonable.

## Discussion

With our system, we were able to present the potential for MEG measurements. Taking into account the effective diameter of the pump and probe beams, the active volume of our magnetometers is approximately ${33}\, \hbox {mm}^{3}$, which is roughly one-tenth of the ${280}\,\hbox {mm}^{3}$ reported in Ref.^21^ and the ${500}\,\hbox {mm}^{3}$ in Ref.^23^. Regarding magnetic field sensitivity, our system achieved about ${16.7}\,\hbox {pT/Hz}^{1/2}$, whereas Reference^21^, utilizing shielding with aluminum and EMS panels, reached ${14.7}\,\hbox {fT/Hz}^{1/2}$ at 300 kHz, and Ref.^23^ achieved ${7}\,\hbox {fT/Hz}^{1/2}$ at 75 kHz with only aluminum shielding. These values are approximately three orders of magnitude more sensitive than our system. Nonetheless, our system focuses on achieving sensitivity to 10 Hz magnetic fields for biomagnetic measurements, despite its modulation frequency being 100 kHz. The difference in performance is likely attributed to 1/f noise, which becomes significant in the lower frequency range. Based on the results obtained within the magnetic shield shown in Fig. 5, the magnetic noise level of our system was ${55}\,\hbox {fT/Hz}^{1/2}$. When compared to the approximately ${2}\,\hbox {fT/Hz}^{1/2}$ obtained in the magnetic shield of Ref.^23^, this value was reasonable considering the effective volume.

We compare the MR images obtained with our system to those from the ultra-low-field OPM-MRI described in Ref.^21^. While the imaging parameters are almost identical, except for a slight difference in TE values, the SNR of the MR images obtained in this study, with NEX = 8, is approximately 7. In contrast, the images from Ref.^21^, with NEX = 16, have an SNR of about 18. Even accounting for the fact that SNR improves in proportion to the square root of NEX, the image quality in Ref.^21^ is about 1.8 times better than that in this study. This result suggests that using OPM for MRI detection could lead to improved SNR, and is consistent with the comparison between OPMs and pickup coils conducted in Ref.^23^. However, the diagonal artifacts seen in Ref.^21^ were not observed in our study, suggesting that our system demonstrates higher stability. Additionally, due to the narrow bandwidth of OPM, there are concerns about resolution degradation when imaging larger areas. In contrast, non-cryogenic pickup coils, which do not suffer from bandwidth limitations, may be more effective when considering expansion to human systems.

**Fig. 10:**
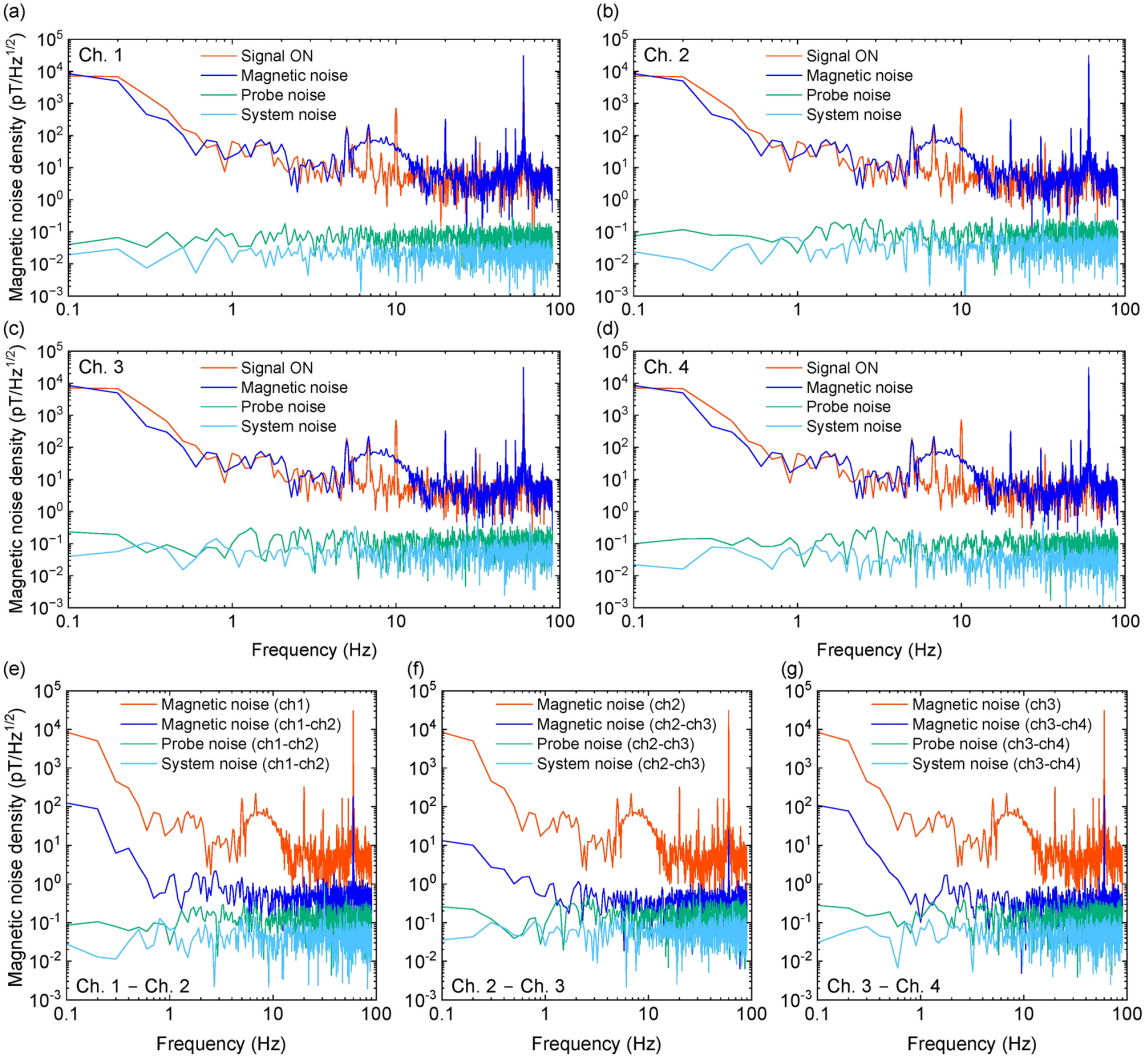
Assessment of impact of the MRI operation on the performance of the scalar-mode OPM. Magnetic noise density at (**a**) Ch. 1, (**b**) Ch. 2, (**c**) Ch. 3 and (**d**) Ch. 4, and differential measurements of each pair of two channels (**e**) Ch. 1–Ch. 2, (**f**) Ch. 2–Ch. 3 and (**g**) Ch. 3–Ch. 4 of the OPM module after MRI operation.

In this multimodal system, where MEG and MRI are intended to be sequentially measured, it is crucial to investigate whether the application of a relatively large magnetic field during MRI impacts MEG measurements. Hence, we conducted a measurement of the magnetic field sensitivity of the OPM module after MRI operation, and the results are illustrated in Fig. 10. Here, after acquiring MR images using the MRI coil set, magnetic noise was measured with the OPM immediately after turning off the current in the coil set. The magnetic noise density of each channel after MRI operation was about ${42}\,\hbox {pT/Hz}^{1/2}$, and MRI operation had resulted in an approximately 2.5-fold increase in noise across all channels. However, in the case of differential measurements, there was a consistent improvement in magnetic noise density for all measurements. This suggests that the significant current flow in the shield wall during MRI operation altered the magnetic field environment within the shield. The consistency of probe noise further supports this observation. Despite fluctuations in the magnetic field environment, the capability of maintaining robust sensitivity through differential measurements positions our newly developed system as a promising MEG and MRI fusion system.

**Fig. 11:**
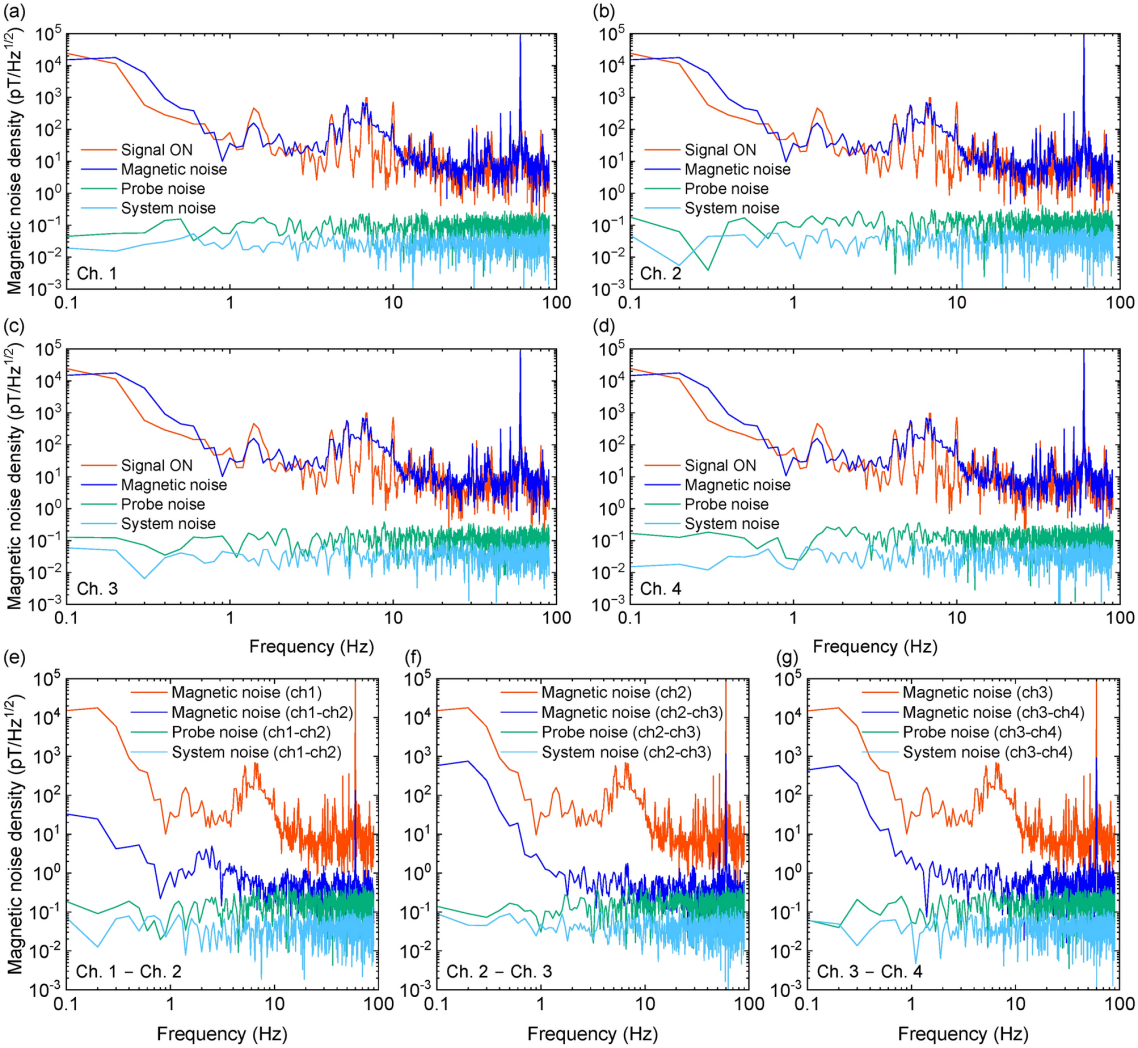
Magnetic Shieldless examination of the scalar-mode OPM. Magnetic noise density at (**a**) Ch. 1, (**b**) Ch. 2, (**c**) Ch. 3 and (**d**) Ch. 4, and differential measurements of each pair of two channels (**e**) Ch. 1–Ch. 2, (**f**) Ch. 2–Ch. 3 and (**g**) Ch. 3–Ch. 4 of the OPM module without magnetic shield.

We examined the operation without any magnetic shields. Figure 11 illustrates the magnetic noise density when the EMS panels used as an magnetic shield was removed. The magnetic noise density for each channel was approximately ${60}\,\hbox {pT/Hz}^{1/2}$. However, through differential measurements, the magnetic noise density could be reduced to about ${370}\,\hbox {fT/cm/Hz}^{1/2}$ for conditions other than Ch. 3–Ch. 4, demonstrating that operation without the magnetic shield is feasible. Nevertheless, in the frequency range below 5 Hz, the noise difference was not discernible, and under Ch. 3–Ch. 4 condition, the magnetic noise density was more than double compared to the shielded case. Therefore, the advanced measures, such as higher-order differential measurements are likely required for measurements without magnetic shields.

The current system has a 140 mm gap between coils, which is too narrow to measure a human head. Therefore, to aim for clinical application, it is necessary to increase the coil spacing. The relationship between gradient field strength and bandwidth becomes an issue in this context. For instance, if the coil spacing is doubled while keeping the gradient field strength and bandwidth fixed, the size of one pixel will also double, leading to a decrease in resolution. Furthermore, if the uniformity of the magnetic field decreases, the measurement sensitivity of the OPM might be compromised due to $T_2^*$ relaxation. Additionally, for MEG, sensors need to be arranged to surround the subject’s head, which necessitates careful attention to the strength and direction of the magnetic field at the sensor positions. To address these issues, it is necessary to thoroughly examine the magnetic field distribution within the system and adjust it to achieve the desired magnetic field distribution using methods such as the target field method^29^.

Another challenge is how to correct for small head movements during measurements. In MEG-MRI systems using SQUIDs, where the sensor positions are fixed, calibration can be performed computationally by utilizing the fixed geometry^30^. However, in the case of on-scalp MEG, where the sensor positions and orientations are not fixed, extra care must be taken when co-registering structural and functional images^14^. Pfeiffer et al. aligned the positions using dipole-shaped coils^31^, while Pang et al. proposed an automatic alignment scheme using a laser scanner^32^. These methods are also applicable to our system, and we plan to consider their implementation in the future.

## Conclusion

We designed and assessed a prototype combining MEG utilizing a scalar-mode OPM module and MRI with non-cryogenic pickup coils, exploring its capabilities. With a simplified magnetic shield, we achieved a noise level of about ${16.7}\,\hbox {pT/Hz}^{1/2}$ with a single channel magnetometer, and reached a noise level of ${367}\,\hbox {fT/cm/Hz}^{1/2}$ through differential measurements. The system successfully conducted MR imaging on a phantom, demonstrating the potential of MEG and MRI fusion. Furthermore, we demonstrated the sustained noise levels in differential measurements both pre and post MRI operation, showcasing the viability of our system as a fusion device. Future challenges encompass advancing sensor performance through enhancements in lasers and optical systems, along with accelerating imaging via sequence improvements in MRI. Our prospective aim involves utilizing this system for actual MEG measurements, progressing towards the realization of MEG and MRI fusion.

## Data Availability

The datasets used and analysed during the current study available from the corresponding author on reasonable request.
